# Short and mid-term follow-up of patients with low ejection fraction who underwent coronary artery bypass surgery: comparison of preoperative and postoperative anginal complaint and functional capacity

**DOI:** 10.1186/1749-8090-8-S1-P101

**Published:** 2013-09-11

**Authors:** M Akyuz, B Lafci, U Yetkin, M Bademci, B Ozpak, I Akyildiz, B Ozcem, A Gurbuz

**Affiliations:** 1 Department of Cardiovascular Surgery, Izmir Katip Celebi University Ataturk Training and Research Hospital, Izmir, Turkey; 2 Department of Cardiology, Izmir Katip Celebi University Ataturk Training and Research Hospital, Izmir, Turkey

## Background

Revascularisation of patients with low ejection fraction has beneficial effects. It prevents progressive left ventricular dilatation, sudden cardiac death, reduction of functional capacity.

## Methods

Thirty-seven consecutive patients with low EF (≤30%) who underwent CABG in our department between February 2010 and April 2012 were analyzed retrospectively (8 women and 29 men, mean age 62,32±10,86 years, range 40 to 78 years). Short and mid term results of CABG on functional capacity and angina were evaluated. Functional capacity and angina were ranked according to the New York Heart Association (NYHA) and Canadian Cardiovascular Society (CCS) classification systems consecutively. Eight patients underwent of-pump.

## Results

In-hospital mortality was 5.4%. One patient died seven months after hospital discharge and mortality was due to heart failure. Annual mortality was 8.1%. The patients did have significantly decreased angina and increased functional capacity after the operation. Sixteen patients were in CCS class I/II and 19 patients were in CCS III/IV preoperatively while all patients were in CCS class I/II postoperatively. Eighteen patients were in NYHA class I/II and 17 patients were in NYHA class III/IV preoperatively while all patients were in NYHA class I/II postoperatively. Postoperative mean values of CCS angina and NYHA functional capacity classifications were significantly lower than preoperative scores (p<0,05). Postoperative mean value of CCS angina class improvement found as 1,35±0,37 and mean value of NHYA functional capacity improvement was 1,33±0,25 when compared to preoperative status. Six and 12-month outpatient CCS angina class and NHYA functional capacity did not show any significant difference.

## Conclusion

Short and mid term follow-up of patients with low EF who underwent CABG, revealed that CCS angina and NYHA functional capacity were improved significantly when compared to their preoperative status.

